# Prosthetic joint and implant contamination caused by *Ralstonia pickettii*: a report of three cases٭

**DOI:** 10.1051/sicotj/2017017

**Published:** 2017-04-11

**Authors:** Brett D. Edwards, Ranjani Somayaji, Bayan Missaghi, Wilson W. Chan, Aaron J. Bois

**Affiliations:** 1 Internal Medicine Residency Program, Department of Medicine, University of Calgary 1403 – 29 St. NW, North Tower, Office 933 Calgary Alberta T2N 2T9 Canada; 2 Section of Infectious Diseases, Department of Medicine, University of Calgary 3330 Hospital Drive NW Calgary Alberta T2N 4N1 Canada; 3 Medical Microbiology, University of Calgary 9-3535 Research Road NW Calgary Alberta T2L 2K8 Canada; 4 Section of Orthopaedic Surgery, Department of Surgery, University of Calgary 3330 Hospital Drive NW Calgary Alberta T2N 4N1 Canada

**Keywords:** Prosthetic joint infection, Contamination, *Ralstonia*, Ultrasonicator, Sonication

## Abstract

We describe three cases of orthopaedic contamination caused by *Ralstonia pickettii* grown from prosthetic joint and implant material cultures following sonication in the microbiology laboratory. Given the temporal association between the cases, lack of clinical or intra-operative features of infection, growth of the organism in the water bath, and unlikely etiology of *Ralstonia* as a prosthetic joint or implant pathogen, the bacteria were judged to be contaminants.

## Introduction

Prosthetic joint infections (PJIs) represent a challenging complication following arthroplasty procedures, and often require a multidisciplinary approach for their optimal management. Such infections typically involve pain at the operative site and may exhibit overt signs of infection on examination and radiographic evidence of loosening. However, low-virulence microorganisms (e.g. *Propionibacterium acnes*) that do not typically evoke a suppurative inflammatory response have become a recognized cause of failed shoulder arthroplasty [1].

**Figure 1.**
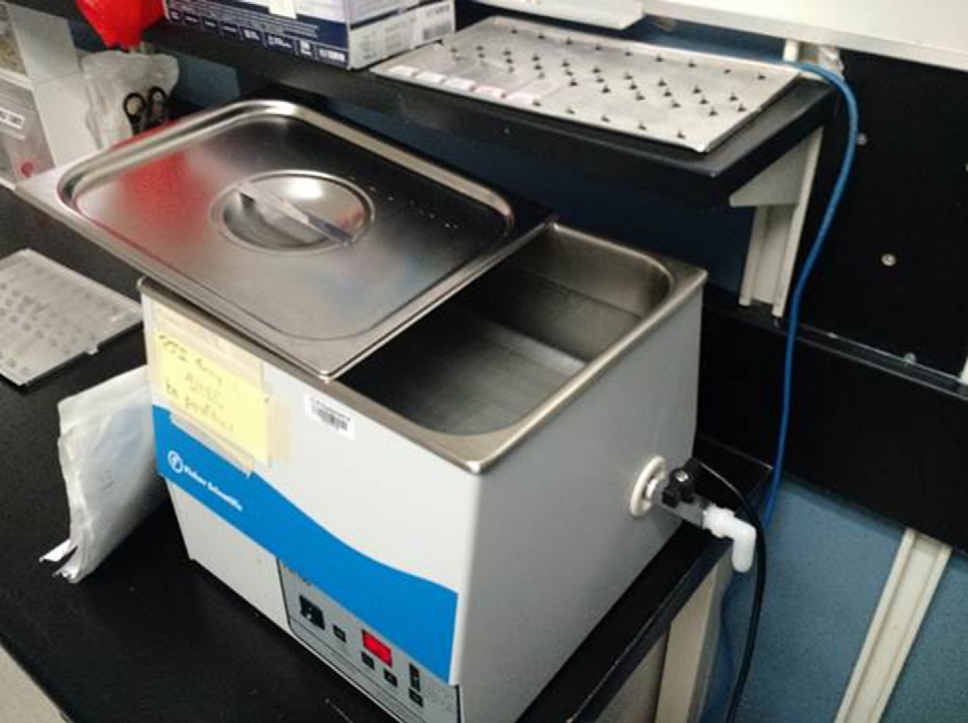
Ultrasonicator device used to disrupt bacterial biofilm on prosthetic and implant devices. The prosthetic device is placed in a container filled with saline and then placed in this waterbath where ultrasound waves are directed at it.

Sonication is increasingly utilized in microbiology laboratories to augment the yield of bacterial culture from prosthetic devices [2]. Explanted prosthetic joints and hardware are placed in a container of saline and submerged in a water bath (Figure 1). Ultrasound waves are directed through the bath at the prosthesis to disrupt existing biofilm, which increases the sensitivity of culture results [3]. Unfortunately, contamination during this process has also been reported [4].

**Figure 2.**
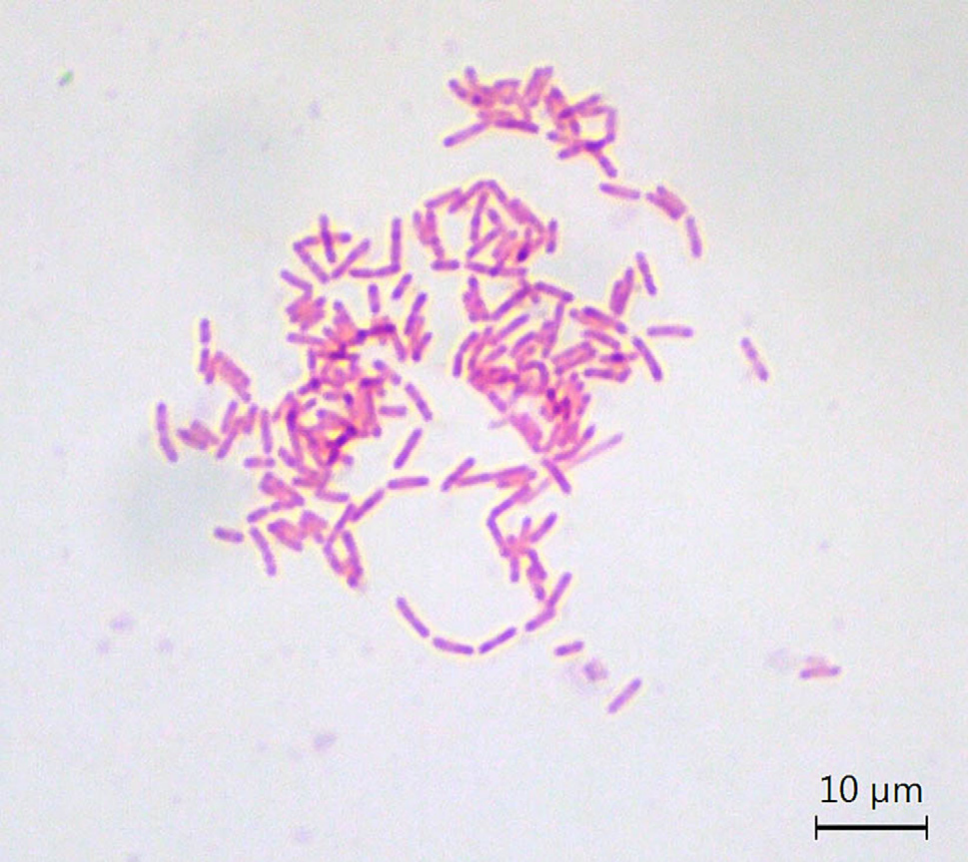
Gram stain of *Ralstonia pickettii* under light microscopy.

*Ralstonia pickettii* has not previously been reported as a causative pathogen in cases of true PJI (Figure 2). Herein, we report three cases of suspected PJI caused by *R. pickettii* and illustrate when contamination should be considered. Each patient provided written consent for their case to be published.

### Case 1

A 38-year old female with degenerative disc disease underwent an elective L4-L5/L5-S1 discectomy followed by total disc replacement. In the acute post-operative period, she was diagnosed with a surgical site infection resulting from *Staphylococcus epidermidis*. She was treated with surgical debridement and antibiotic therapy for nine months with symptom improvement. Three months after antibiotics were discontinued, she presented to hospital with fever and progressive back pain. Bloodwork revealed hemoglobin 121 g/L, white blood cells (WBC) 7.6 × 10^9^/L, platelets 193 × 10^9^/L, and C-reactive protein (CRP) 9.0 mg/L. Diagnostic imaging revealed improvement in the previous site of infection, but suggested a potential new focus of infection adjacent to the prosthetic disc. The patient subsequently underwent the first stage of a planned two-stage revision disc arthroplasty. One deep-tissue specimen and two prosthetic implants were sent for bacterial and fungal cultures.

Both the tissue and the L4-L5 prosthesis cultures were negative after extended incubation; however, the L5-S1 prosthesis grew scant *R. pickettii* after two days’ incubation. The patient was initiated on broad-spectrum antibiotic therapy for possible residual infection. After six days (as the events detailed below in Case 2 were occurring), the laboratory informed the treating surgeon that the *Ralstonia* likely represented contamination. Accordingly, the patient was discharged home on vancomycin and rifampin for six weeks’ duration to treat her presumed recurrence of *S. epidermidis*. After two years of follow-up she has had no evidence of recurrent infection.

### Case 2

A 70-year-old female with multiple medical comorbidities presented with a history of periprosthetic femur fracture complicated by infection that was treated with an excision hip arthroplasty, antibiotic cement spacer, and parenteral antibiotics. Reimplantation was delayed for unrelated medical concerns. At the time of the second stage, she was medically stable with normal bloodwork and imaging. No intra-operative purulence was noted.

Four deep-tissue specimens and the antibiotic spacer were sent for bacterial and fungal cultures. All tissue cultures were negative; however, the spacer grew scant *R. pickettii* after two days’ incubation. Given the temporal proximity to Case 1, an investigation in the laboratory was undertaken. The sonication process, which differentiated the culture of the prosthesis from that of the tissues, was hypothesized to be responsible. Culture of the sonicator water bath grew > 10^5^ CFU/mL of *R. pickettii*. During sonication, water from the bath likely penetrated the container enclosing the prosthesis, contaminating its contents. Pulsed-field gel electrophoresis (PFGE) suggested that the *Ralstonia* isolates from Cases 1 and 2 were identical [5]. Based on the patient’s clinical presentation and likelihood of laboratory contamination, no antibiotic therapy was initiated and she remained well at latest follow-up.

### Case 3

A 76-year-old female with a history of marginal zone lymphoma incurred ipsilateral displaced fractures of her left proximal humerus and proximal ulna after a fall. She underwent open-reduction and internal fixation (ORIF) to stabilize both fractures (performed by the senior author [AJB]).

The patient developed progressive shoulder pain four months following surgery without features of infection. Radiographs revealed subtle lucency around the distal screws and delayed union of the surgical neck resulting from aggressive postoperative physical therapy. The rehabilitation program was modified, yet her symptoms persisted. Eighteen months after the index operation the patient underwent surgical debridement, revision ORIF, and bone grafting of a surgical neck nonunion. There were no intra-operative features of infection. She was clinically stable and preoperative bloodwork revealed hemoglobin 100 g/L, WBC 12.9 × 10^9^/L, platelets 344 × 10^9^/L, and CRP 2.9 mg/L.

**Figure 3.**
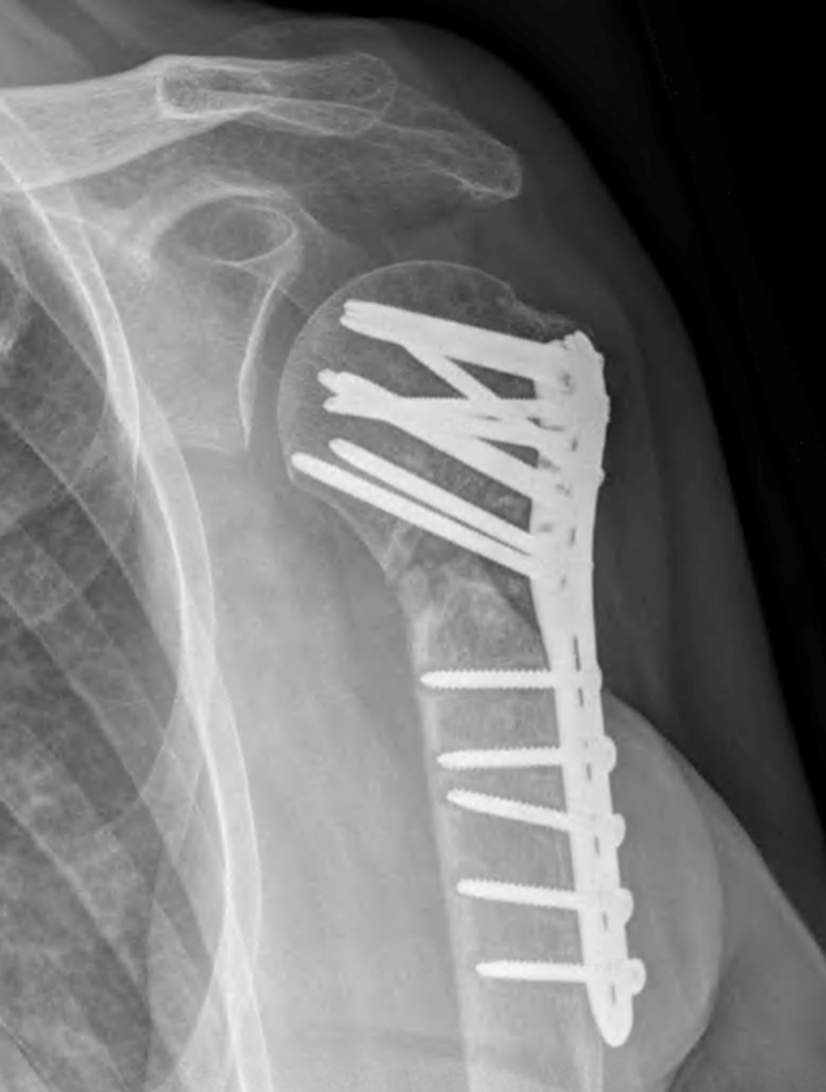
True AP view of the left shoulder approximately 15 months from undergoing revision ORIF and bone grafting of a surgical neck nonunion of the proximal humerus. There is evidence of early hardware failure (screw pullout), graft resorption, and persistent nonunion of the surgical neck.

Multiple deep-tissue cultures and the humeral plate were sent for microbiological and pathological assessment. Histopathology revealed chronic inflammation with rare neutrophils and no bacteria. Bacterial culture of the plate grew *R. pickettii* after three days’ incubation. However, the laboratory notified the treating physicians that contamination was likely based on the previously reported cases. Culture of the water bath again grew *R. pickettii*. All other culture results were negative including those specifically held for *P. acnes* (i.e., after 14 days). At 15 months of follow-up, the patient complained of worsening shoulder pain but no infectious symptoms. Radiographically, she was found to have a persistent atrophic nonunion of the surgical neck with early hardware loosening (Figure 3). Preliminary infection work-up was normal (i.e., bloodwork and WBC scan) while metabolic work-up revealed Vitamin D deficiency. Plans were made for revision surgery.

## Discussion

This case series describes three patients with suspected prosthetic joint/hardware infection with *Ralstonia pickettii* that were deemed to be contaminants. *Ralstonia* species are aerobic, Gram-negative rods that are typically opportunistic pathogens [6]. Strains have been isolated from hospital water supplies, sinks, and bronchoscopes and may cause diverse infections with reports of endocarditis, osteomyelitis, respiratory infection, and meningitis [7]. Infections are seen more frequently in immunocompromised hosts including those with hematologic malignancies [8]. *Ralstonia* species have also been implicated in intravascular catheter systems as these bacteria readily form biofilm. The removal of foreign material is often required for cure [6]. Rarely detected in clinical specimens, *Ralstonia* is often a contaminant with predilection for water and has been reported as an agent of pseudo-outbreaks. Verschraegen et al. reported 17 spurious “bacteremias” from contaminated hospital chlorhexidine solution [9]. More recently, a *Ralstonia* pseudo-outbreak occurred through contamination of a disinfectant used for blood culture bottles [8].

Prosthetic joint infections occur in up to 4% of primary hip, knee, and shoulder arthroplasties and at greater frequency in revision arthroplasties [10–12]. Only 11–23% of PJIs are attributed to Gram-negative bacteria; such infections may be particularly challenging due to increased virulence and antimicrobial resistance [13]. *Pseudomonas aeruginosa*, *Escherichia coli*, and *Klebsiella* species are the most common Gram-negative organisms associated with PJI. A prosthetic joint infection resulting from *Ralstonia* has not been previously reported; one case has been reported where *Ralstonia* was unexpectedly cultured following a revision elbow arthroplasty, however, this was considered a contaminant and was successfully managed without further treatment [14].

By disrupting biofilms with ultrasound waves, sonication increases the detection of bacteria on explanted devices aiding in the diagnosis of PJI [2]. Conversely, sonication requires additional manipulation of the specimen, increasing the opportunity for contamination [4]. In a previous report, a collection of organisms that included *Ralstonia* species, were incidentally identified in patients with aseptic prosthesis loosening following sonication and PCR. The pathogenicity of *Ralstonia* in this series was inconclusive [10].

In our series, *R. pickettii* was cultured from the water bath used for sonication, which was the most probable source of contamination. The containers housing the hardware were not watertight, leading to contamination of the sterile contents. Pulsed-field gel electrophoresis linked the *Ralstonia* isolates from Cases 1 and 2, suggesting a common source.

Following Case 2, laboratory measures to stop contamination were implemented including weekly cleaning of the water bath, sterile transfer of contents in and out of the sonication containers, and sealing the containers with Parafilm while sonicating. Subsequently, no cases of contamination occurred for 1.5 years. Following Case 3, an investigation revealed the sealing step was neglected. The importance of this step was reiterated to frontline staff and no further *Ralstonia* prosthetic joint/hardware associated cases have occurred.

When an opportunistic pathogen of low virulence such as *Ralstonia* is identified in cases of PJI, differentiating contaminant from true pathogen must be based on the clinical context. Supporting information may include clinical signs and symptoms of infection, biochemical tests (i.e. WBC and platelet count, erythrocyte sedimentation rate [ESR], CRP), diagnostic imaging, and intra-operative findings such as purulence. In addition, the clinician should consider the pathogenicity of the organism in question and the location of recovery. For instance, *R. pickettii* is frequently isolated in nosocomial water supplies. Finally, one must consider contamination when there are multiple cases of common contaminating bacteria over a short period, particularly in patients that have no shared epidemiologic or clinical risk factors. Collectively, these factors aid in the proper diagnosis of bacterial contamination and prevent unnecessary revision surgery and patient morbidity.

In this study, we highlight the potential for bacterial contamination of prosthetic material at any stage of culture and the importance of supporting clinical, radiographic, and operative information in the optimal management of PJI.

## Conflict of interest

BE, RS, BM, WC, AB certify that they have no financial conflict of interest (e.g. consultancies, stock ownership, equity interest, patent/licensing arrangements, etc.) in connection with this article.

The authors did not receive any outside funding or grants in support of their research for or preparation of the work. Neither they nor a member of their families received payments or other benefits or a commitment or agreement to provide such benefits from a commercial entity.
